# Pistil Mating Type and Morphology Are Mediated by the Brassinosteroid Inactivating Activity of the *S*-Locus Gene *BAHD* in Heterostylous *Turnera* Species

**DOI:** 10.3390/ijms221910603

**Published:** 2021-09-30

**Authors:** Courtney M. Matzke, Hasan J. Hamam, Paige M. Henning, Kyra Dougherty, Joel S. Shore, Michael M. Neff, Andrew G. McCubbin

**Affiliations:** 1 School of Biological Sciences, Washington State University, P.O. Box 644236, Pullman, WA 99164-4236, USA; courtney.matzke@wsu.edu (C.M.M.); paige.henning@wsu.edu (P.M.H.); 2 Department of Biology, York University, 4700 Keele Street, Toronto, ON M3J 1P3, Canada; hasanhamam12@gmail.com (H.J.H.); kyrad@my.yorku.ca (K.D.); shore@yorku.ca (J.S.S.); 3 Department of Crops and Soils, Washington State University, P.O. Box 644236, Pullman, WA 99164-6420, USA; mmneff@wsu.edu

**Keywords:** heterostyly, self-incompatibility, brassinosteroid, BAHD acyltransferase

## Abstract

Heterostyly is a breeding system that promotes outbreeding through a combination of morphological and physiological floral traits. In *Turnera* these traits are governed by a single, hemizygous *S*-locus containing just three genes. We report that the *S*-locus gene, *BAHD*, is mutated and encodes a severely truncated protein in a self-compatible long homostyle species. Further, a self-compatible long homostyle mutant possesses a *T. krapovickasii* *BAHD* allele with a point mutation in a highly conserved domain of BAHD acyl transferases. Wild type and mutant *TkBAHD* alleles were expressed in *Arabidopsis* to assay for brassinosteroid (BR) inactivating activity. The wild type but not mutant allele caused dwarfism, consistent with the wild type possessing, but the mutant allele having lost, BR inactivating activity. To investigate whether BRs act directly in self-incompatibility, BRs were added to in vitro pollen cultures of the two mating types. A small morph specific stimulatory effect on pollen tube growth was found with 5 µM brassinolide, but no genotype specific inhibition was observed. These results suggest that *BAHD* acts pleiotropically to mediate pistil length and physiological mating type through BR inactivation, and that in regard to self-incompatibility, BR acts by differentially regulating gene expression in pistils, rather than directly on pollen.

## 1. Introduction

The benefits associated with genetic diversity have driven the evolution of an extraordinary array of mechanisms that promote outbreeding in the eukaryotes. In the flowering plants, though single sex individuals are found in some species, echoing gonochorism in animals, most are hermaphrodite [1]. Within flowers, male and female organs often mature in close proximity, yet it has been estimated that ~50% of angiosperm species obligately outbreed [2]. Mechanisms that have evolved to promote outbreeding include spatial and/or temporal separation of mature male and female organs, and biochemical mechanisms that recognize and reject self or genetically related gametes (known as self-incompatibility systems) [3,4]. Heterostyly is a breeding system characterized by mating types with morphological characteristics that encourage cross-pollination, often combined with biochemical incompatibilities that prevent within-morph fertilization. In species with heterostyly, individuals are one of two (distyly) or three (tristyly) distinct floral morphs and male and female organs are reciprocally positioned (within flowers) between mating types. In distyly this is manifested as a short styled (S-) morph with long stamens, and a long styled (L-) morph with short stamens. Intriguingly heterostyly is polyphyletic and appears to have independently evolved many times in the angiosperms [5]. This combination of characteristics has fueled scientific interest and considerable efforts to unravel the evolution of, and genetic mechanisms underpinning, heterostyly for ~150 years.

The function of the floral forms in contributing to outcrossing in heterostyly was first proposed by Darwin [6]. This work also reported the occurrence of rare natural homostyle individuals of otherwise heterostylous species, which have flowers with juxtaposed stamen and stigma, either “long” homostyles with long-styles and long stamens, or “short”homostyles with short-styles and short stamens [6]. In both long and short homostyles the physiological mating type of either the pistil or the stamen is switched (as well as the morphology), such that male and female organs within a flower exhibit different mating types and as a result self-compatibility [6]. The inheritance of homostyly became a popular genetic model after the rediscovery of Mendel’s work in the early 20th C. In particular data for *Primula* species collected over more than 30 years by Ernst is integral to our understanding of the genetics of heterostyly [7]. Ernst and later authors who reanalyzed his data proposed heterostyly to be controlled by a supergene locus, termed the *S*-locus [7,8,9]. This *S*-locus was hypothesized to possess just two alleles (*S* and *s*), the short styled S-morph being heterozygous, *Ss*, and the long styled L-morph homozygous recessive, *ss* [9,10]. Genetic analyses of progeny resulting from crosses between homostyle and wild type morphs suggested at least three recombinable sub-units within the *S* linkage group: *G* (female gynoecium characteristics), *P* (pollen size) and *A* (male androecium characteristics) [7,8]. Though this interpretation of the subcomponents of the *S*-locus still stands, recent molecular data have necessitated reevaluation of the genetics of heterostyly [11]. Heterostylous species from the 3 unrelated genera examined to date, which are believed to have evolved heterostyly independently, all possess *S*-loci that are hemizygous in the S-morph but absent in the L-morph. Further in all cases assessed at the molecular level, homostyly has been determined to result from mutation of individual genes within the *S*-locus (*S*-genes) rather than recombination between alleles as previously proposed [11]. That the *S*-loci that control heterostyly are hemizygous in one mating type and absent in the other is thought provoking, as it suggests that these mating type loci in plants share functional and physical commonalities with animal sex chromosomes, albeit that they control breeding systems that retain hermaphrodite mating types.

The genes that reside within *S*-loci and control distyly have been identified in several unrelated genera, including *Primula*, *Turnera,* and *Fagopyrum*. The roles and modes of action of these *S*-genes are focal points of current research. In *Primula*, five *S*-genes have been identified: *CYP734A50* (a cytochrome p450)*,* GLOBOSA2 (*GLO2*, a MADS box transcription factor)*, PUM* (a Pumilio-like RNA-binding protein), *CCM* (a cyclin-like F box gene), and *KFB* (a Kelch repeat F box protein) [12,13,14,15,16]. *CYP734A50* is expressed in pistils and is a member of the P450 CYP734A cytochrome family [14]. This gene controls pistil length in the S-morph by degrading brassinosteroids (BRs), leading to reduced cell expansion [14]. The MADS-box transcription factor *GLOBOSA2* (*GLO2)* is stamen specific [12,13,16], and controls anther height by promoting cell expansion below the point of anther filament insertion in the corolla tube [15]. Potential functions for the other three *Primula S*-genes have not yet been identified. In *Fagopyrum*, style length is regulated by *S-*locus early flowering 3 (*S-ELF3*), a homolog of *Arabidopsis* *thaliana ELF3,* a nuclear protein that interacts with phytochrome B to control plant development and flowering [17,18].

The *Turnera S*-locus has been fully sequenced in *T. subulata* and possesses just three *S*-genes [19]. Two are expressed in stamen, *TsSPH1* (homologous to *Papaver rhoeas* stigma incompatibility *S-protein*) and *TsYUC6* (homologous to the *YUCCA* gene family of flavin-dependent monooxygenases)*. TsSPH1* is expressed in anthers and stamen filaments, and proposed to control filament length [19]. *TsYUC6*, which is expressed only in anthers, has been proposed to control pollen incompatibility mating type through auxin synthesis [19]. The third *S*-gene, *TsBAHD*, is the sole *S*-gene expressed in pistils and has conserved motifs found in BAHD acyltransferases [19,20]. The closest characterized *A. thaliana* homologs of *TsBAHD* include *BIA1*, a gene reported to acetylate and inactivate BRs [21]. When over-expressed in *A. thaliana*, *BIA1* causes a phenotype that mimics mutants defective in BR synthesis, severe dwarfism, shortened stems and petioles, small epinastic, dark green leaves, delayed senescence, and infertility [21]. We recently took advantage of this phenotype to use *A. thaliana* as a bioassay to confirm that *TsBAHD* possesses BR inactivating activity [20]. As the only *S*-gene expressed in pistils, *BAHD* is predicted to control all female characteristics, namely pistil length and female biochemical mating type. However, these predictions have not been empirically confirmed, and whether BR inactivating activity is the mode of action by which *BAHD* mediates these traits remains to be demonstrated.

In this study, we characterize *BAHD* in a self-compatible long homostyle species, *T. campanifolia*, and wild type and a spontaneous self-compatible long homostyle mutant allele of the heterostylous species *T. krapovickasii*. We show that both self-compatible homostyles carry mutant *BAHD* alleles. In *T. campanifolia* mutation of *BAHD* results in severe truncation of its protein product. In *T. krapovickasii* a single point mutation in a conserved domain was identified in the homostyle, relative to the wild type *BAHD*, allele. Both wild type and mutant *TkBAHD* alleles were assayed for their ability to inactivate brassinosteroids using an *Arabidopsis thaliana* bioassay. Expression of wild type *TkBAHD* in *A. thaliana* caused dwarf phenotypes indistinguishable from those previously reported for *BAHD* from *T. subulata* [20]. In contrast, expression of the mutant allele caused little if any reduction in growth, consistent with the point mutation dramatically reducing the ability of the protein to inactivate BRs by acylation. These results are consistent with *BAHD* being the *S*-gene that controls both pistil length and female mating specificity in *Turnera* and BR mediating these traits.

## 2. Results

### 2.1. Analysis of BAHD from Homostyle Species

**Figure 1:**
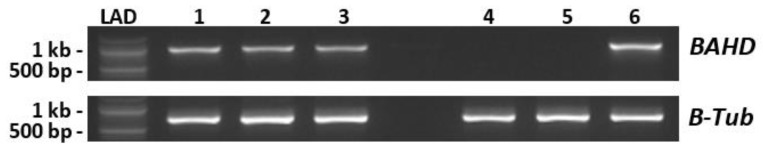
Assessment of *BAHD* expression in styles of long homostyle species. Lanes 1–3. RT-PCR of three individuals of *T. campaniflora*, using primers BAHD-1F and BAHD1022R (Table S1). Lanes 4–5. two individuals of *T. ulmifolia.* Lane 6. *T. subulata* S-morph (positive control). Upper panel–*BAHD*. Lower panel–*β-Tubulin*. LAD, 100 bp ladder.

Using primers designed to conserved regions of BAHD acyl transferases, we attempted to amplify *BAHD* from pistil cDNA of *T. campanifolia* and *T. ulmifolia* (long homostyle species bearing flowers with L-morph female and S-morph male mating types). RT-PCR products of the predicted size were amplified from all individuals of *T. campanifolia*, but none of *T. ulmifolia* (Figure 1). Full length *BAHD* was then amplified from two *T. campanifolia* individuals using primers BAHD-36F and BAHD1513R (Table S1) which flank the entire coding region and the PCR products sequenced directly using three forward and three reverse primers (sequencing primers listed in Table S1).

**Figure 2:**
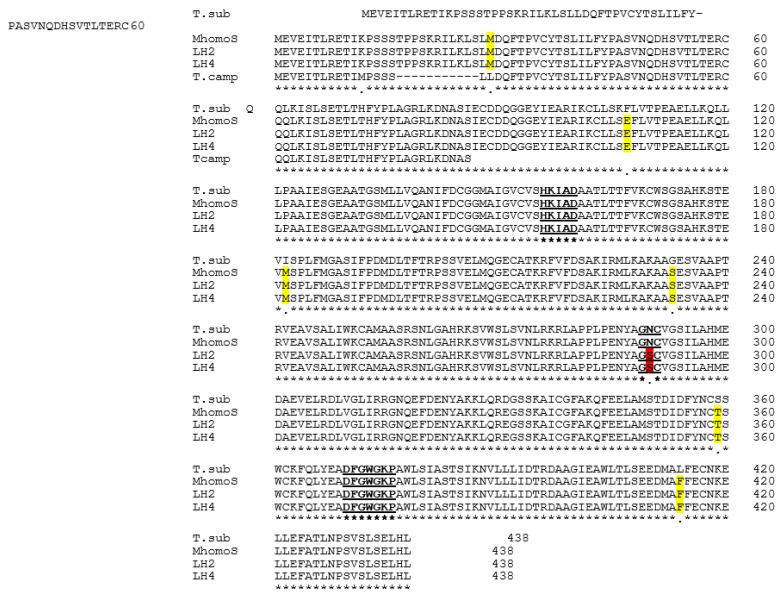
Alignment of *BAHD* amino acid sequences. T.sub, *T. subulata*; MhomoS, S-morph (Genbank accession # MZ018119); LH2 (734H) and LH4 (HH12) long homostyle progeny (Genbank accessions #’s MZ018117 MZ018118); T.camp *T. campanifolia* (homostyle species) (Genbank accession #s MW929184 and MW929185). Regions highlighted in bold and underlined indicate the three conserved regions important for *BAHD* function: HxxxD, GN, and DFGWG motifs. Regions highlighted in yellow indicate amino acid differences between BAHDs of *T. krapovickasii* and *T. subulata*. The amino acid colored red indicates the asparagine (N) residue in the conserved GN motif present in BAHD from plants of *T. subulata* and the S-morph Mhomo-S, but mutated to serine (S) in both long-homostyle plants.

Alignment of the *T. campanifolia* sequences with *BAHD* of *T. subulata*, indicated that the *T. campanifolia* sequences were identical to each other and shared 98% identity with *TsBAHD* at the nucleotide level. A 33 bp indel was identified towards the 5′ end of the *TcBAHD* sequence that causes a loss of 11 amino acids (but does not alter the reading frame) in the predicted protein relative to *TsBAHD*. A smaller, but more functionally significant difference was a single bp deletion in *TcBAHD* at a position equivalent to base 357 of *TsBAHD*, this deletion results in a frameshift that immediately introduces a stop codon, severely truncating the predicted protein (Figure 2). These results suggest that *T. campanifolia,* a self-compatible long homostyle species, is unable to synthesize the functional BAHD enzyme, consistent with BAHD being responsible for short styles and self-incompatibility in heterostylous species. However, the possibility that other genes in *T. campanifolia* are mutated relative to heterostylous species could not be excluded. The failure to detect a product in *T. ulmifolia* is consistent with the absence of *BAHD* in this species, but we cannot rule out the possibility that this result may be due to sequence divergence at primer annealing sites.

### 2.2. S-Gene Expression in the Long-Homostyle Mutant Mhomo-H

The long-homostyle mutant, Mhomo-H has been previously described [22]. This plant arose as a sport-shoot on an otherwise short styled *T. krapovickasii* × *T. subulata* diploid hybrid plant and has been maintained by vegetative propagation. Homostyly in this plant segregates with the *S*-locus and the *S*-allele which it carries termed *S_H_*, giving the genotype *sS_H_* [22]*. S_H_* has been hypothesized to possess a recessive mutant form of the G gene of the *S*-locus complex (which governs pistil characteristics). Surprisingly Mhomo-H periodically produces normal S-morph flowers, hereafter referred to as Mhomo-S, and hence appears to retain a functional *S*-allele though its effects are not always present. Sexual progeny have also been raised from seed produced by selfing and crossing Mhomo-H to an L-morph, with long homostyle, L-morph and very infrequent S-morph phenotypes all found amongst the offspring [22]. Similarly, crosses of an S-morph revertant to an L-morph gave S-morph, L-morph and very infrequent long homostyle progeny. The “reversion” to S-morph flowers is only a characteristic of the original Mhomo-H mutant, flowers of homostyle progeny are phenotypically stable and never show an S-morph phenotype.

**Figure 3:**
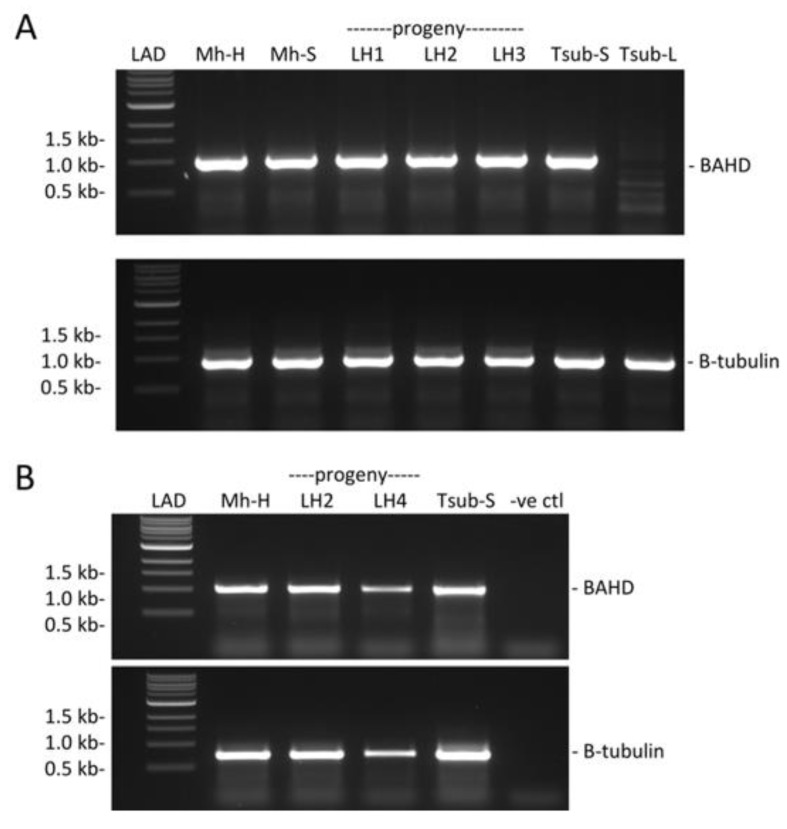
Presence and expression of *BAHD* in long-homostyle mutant Mhomo-H and its progeny. (**A**) gDNA PCR amplification of *BAHD* using gene specific primers. Upper panel, presence of *BAHD* (primers BAHD-1F and BAHD1022R [Table S1]). Samples: Mh-h, long-homostyle mutant (Mhomo-H). Mh-S, S-morph revertant (Mhomo-S). LH1, long-homostyle progeny 1 (859H). LH2, long-homostyle progeny 2 (867H), LH3, long-homostyle progeny 3 (275H). T-sub-S, *T. subulata* S-morph (F60SS), T-sub-L, *T. subulata* L-morph (D16L). LAD: 1kb ladder. (**B**) RT-PCR of style RNA. Upper panel, *BAHD* amplified with gene-specific primers BAHD1F and BAHD1022R, Table S1), lower panel *β-Tubulin* (amplified with primers Tub1F and Tub1R [Table S1]). Samples: Mh-H, Mhomo-H; LH2 and LH4* long homostyle progeny 867H and HH12; Tsub-S, *T. subulata* S-morph; -ve ctl, no cDNA negative control. LAD, 1 kb ladder. *LH4 shows reduced signal for both *BAHD* and *β-Tubulin* as a result of reduced cDNA input due to lack of available tissue.

We used genomic PCR to investigate whether *BAHD* was present in Mhomo-H, its sexual progeny exhibiting long homostyly and Mhomo-S (Figure 3A). *BAHD* amplified from all samples (including the long homostyle mutants) except the L-morph *T. subulata* negative control, suggesting that homostyly in Mhomo-H is not caused by complete deletion of *BAHD*. We next assessed whether homostyly segregating with the *S_H_* allele was caused by lack of *BAHD* expression. Using RT-PCR, *BAHD* was determined to be expressed in all 3 long-homostyle plants tested (Figure 3B). Hence, the long-homostyle phenotype appeared not to be a caused by a lack of *BAHD* expression.

### 2.3. BAHD Sequence Analysis

To investigate whether mutation of *BAHD* might underlie homostyly in Mhomo-H, *BAHD* was amplified by genomic PCR from two long-homostyle progeny derived from Mhomo-H and the revertant S-morph Mhomo-S. PCR products were directly sequenced and assembled as above. The sequences were translated in silico and predicted amino acid sequences aligned to *BAHD* from *T. subulata* (Figure 2). The four *Turnera* sequences possessed three regions conserved among all BAHD family members that have been identified as being essential for interacting with substrates: the HxxxD, GN, and DFGWGKF motifs (Figure 2, [23,24]). Six amino acid differences were identified between the new sequences and TsBAHD, suggesting that the sequences from the long homostyle progeny and Mhomo-S represent *BAHD* from the *T. krapovickasii* rather than the *T. subulata* parent of Mhomo-H. Five of these differences were consistent between all TkBAHD sequences and all are conserved amino acid substitutions. A single amino acid differed between sequences from homostyle and S-morph samples, this being amino acid 290, located within the typically conserved GN motif. In S-morph progeny and *T. subulata*, codon 290 encodes asparagine (AAT), the amino acid conserved with all other BAHD family members, but the sequences from long homostyle plants encoded serine (AGT) at this position disrupting the GN motif. As this mutation lies in a conserved domain, these results suggested the possibility that homostyly in the mutant lines might be caused by a loss of catalytic activity, the single point mutation in *BAHD* leading to a loss of ability to inactivate brassinosteroids.

### 2.4. Does the Mutant BAHD in Mhomo-H Possess Brassinosteroid Activity?

**Figure 4:**
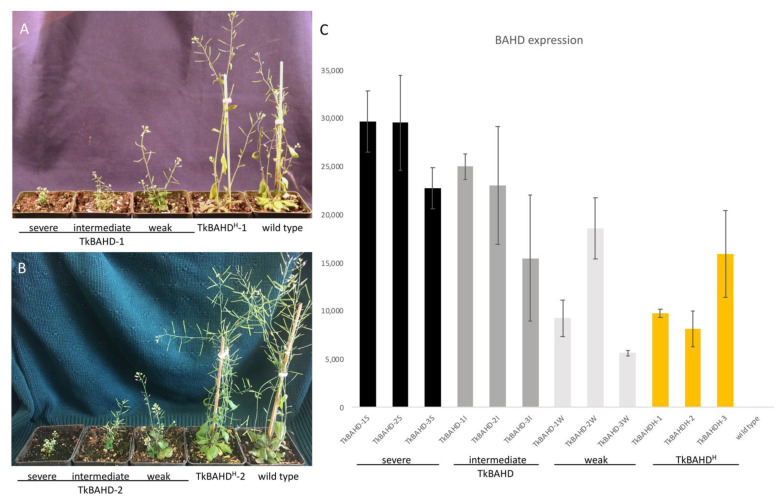
Analysis of *A. thaliana 35S::TkBAHD* and *35S::TkBAHD^H^* transformants. (**A**,**B**), from left to right: phenotypes of six week old *35S::TkBAHD* transformants (TkBAHD-1 and -2) showing weak, intermediate, and severe dwarf phenotypes, and *35S::*TkBAHD^H^ lines -1 (**A**) and -2 (**B**) compared to wild type (Col-0). (**C**) Expression levels (relative to actin) of *BAHD* transgenes in three *35S::*TkBAHD lines, each divided into S, I, and W dwarf categories and *35S::*TkBAHD^H^ lines 1–3, wild-type samples were included as a negative control. Expression was normalized to the actin 8 (At1g49200) housekeeping gene. Error bars represent standard error.

Ectopic expression in *Arabidopsis thaliana* has been used a bioassay for brassinosteroid inactivating activity [25] and we recently used this approach to demonstrate that the acyltransferase encoded by *TsBAHD* possesses this activity [20]. We employed this bioassay to investigate whether the mutation in the GN motif in the allele associated with homostyly (*TkBAHD^H^*) caused a loss brassinosteroid inactivating activity relative to the wild type allele. Each allele was cloned into a plant transformation vector behind the 35S promoter and transformed separately into *Arabidopsis thaliana* by floral dip. Transformants were identified based on kanamycin resistance and the presence of transgenes confirmed by PCR. Three independent lines were selected, assessed by segregation analyses to possess single locus insertions, then homozygous lines (T_4_) generated for each. Consistent with our previous analysis of *TsBAHD* [20], all three lines of wild type *TkBAHD*, displayed dwarf phenotypes, but despite being homozygous single locus insertions, exhibited variation in the severity of dwarfism exhibited. Transformants were grouped based on whether they displayed a severe (S), intermediate (I), or weak dwarf (W) phenotype (Figure 4A,B). Severe dwarfs were very short in stature with very small curled leaves, and extremely reduced inflorescences that produced very little seed on self-pollination. Intermediate dwarfs had larger leaves and flower stalks, and increased seed set compared to the S dwarfs. The weak dwarfs (displaying the mildest phenotype) had slightly paler, larger green leaves and longer inflorescences, but were still substantially reduced in size relative to wild-type controls (Figure 4A,B). These phenotypes are typical of BR-deficient mutants [26,27,28,29] and are indistinguishable with those identified for *TsBAHD* [20]. Phenotypic variability was also previously observed in transformants of *TsBAHD* and is likely a result of transgene suppression [20]. To confirm that the dwarfism observed in plants transformed with *TkBAHD* was caused by altered active BR levels, we assessed expression of genes involved in BR homeostasis. Two genes involved in BR biosynthesis (*DWF4* and *CPD*) were determined to exhibit significantly higher levels of expression in the *TkBAHD* expressing lines, whereas a gene involved in BR inactivation (*BAS1*) showed significantly decreased expression levels relative to wild type plants (Figure S1), as expected for plants deficient in active BRs [20]. These results are consistent with wild type *TkBAHD*, which possesses the ability to determine the S-morph pistil phenotype (short pistils and S-morph pistil mating type) possessing BR inactivating activity as was previously observed for *TsBAHD* [20]. In contrast to the wild type allele, transgenic lines of the mutant allele *TkBAHD^H^* did not exhibit appreciable dwarfism relative to wild type plants (Figure 4A,B).

To confirm that the lack of phenotype for *TkBAHD^H^* transformants was not due to inadequate transgene expression, *TkBAHD^H^* expression levels were assessed by qPCR. For wild type *TkBAHD,* RT-qPCR analysis showed that the severity of dwarfism correlated with relative levels of *TkBAHD* expression, expression generally being highest in S dwarfs and lowest in W. Levels of transgene expression observed for *TkBAHD^H^* were generally lower than those found for the severe and intermediate *TkBAHD* dwarfs, but critically were as high or higher than those observed in weak dwarfs (and in one instance an intermediate dwarf) (Figure 4C). A likely reason that the *TkBAHD^H^* transgenic lines studied expressed lower levels of the transgene than the *TkBAHD* lines is that, for the wild type allele selection for high expression levels based on visible dwarfism occurred, but in contrast, in the absence of a phenotype this selection did not occur for lines expressing the mutant allele. Nonetheless, *TkBAHD^H^* lines expressed as highly as the weaker expressors of *TkBAHD*, but did not exhibit the still substantial dwarfism observed in these lines, suggesting that the mutation identified in *TkBAHD^H^* has caused loss of, or at least a substantial reduction in, BR inactivating activity relative to the wild type protein. Overall these results are consistent with the hypothesis that the BR inactivating activity of BAHD mediates both short style length and physiological mating type in the S-morph of *Turnera*.

### 2.5. Do BRs Act Directly on Pollen to Mediate Self-Incompatibility in Turnera?

The results presented above suggest that the abundance of active BRs mediate physiological mating type in *Turnera* pistils, active BRs being high in L-morph pistils and relatively low in S-morph pistils. There are two potential mechanisms by which this might occur. First BRs have the ability to directly affect pollen germination and tube growth [30], hence presence/absence of active BR could potentially affect pollen germination and/or tube growth of the mating types differentially, creating a mechanism of incompatibility. BRs also have the potential to affect gene expression, however, and as we have previously reported there is considerable differential gene expression between L- and S-morph pistils necessarily caused by the difference in the abundance of active BRs [31], hence BR mediated differential gene expression in the pistils could also mediate mating type. To discriminate between these two possibilities, we assessed the effects of adding BRs to *Turnera* pollen germinated in vitro. Though brassinolide (BL) is the most bioactive BR in plants, variation in the exact BR that is most significant in particular tissues has been reported. For example, castasterone has been postulated to be the major active brassinosteroid during vegetative growth, whereas BL may play an organ-specific role in fruit development in tomato [32]. Not knowing which might be most significant in floral tissues of *Turnera*, we assessed the effects of both BL and castasterone, and germinated and cultured pollen from L- or S-morphs of *T. joelii* (a heterostylous species which possesses the same *S*-genes as those under study [19] and produces a large number of flowers daily, making it a useful species for physiological studies of pollen) in media supplemented with each. If the hypothesis that active BR’s act directly on pollen in self-incompatibility is correct, it is predicted that S-morph pollen germination and/or tube growth would be inhibited under low BR conditions (mimicking self-pollination of an S-morph, low BR pistil), whereas L-morph pollen would be unaffected or show promotion of germination and/or growth. Conversely under high BR conditions S-morph pollen would be unaffected or show growth promotion and L-morph pollen would be inhibited (as L-morph pistils have relatively high active BR concentrations because they do not possess BAHD). Hence, germination or growth was compared to the controls (lacking exogenous BR) within each morph to test for positive or negative effects of BRs.

**Figure 5:**
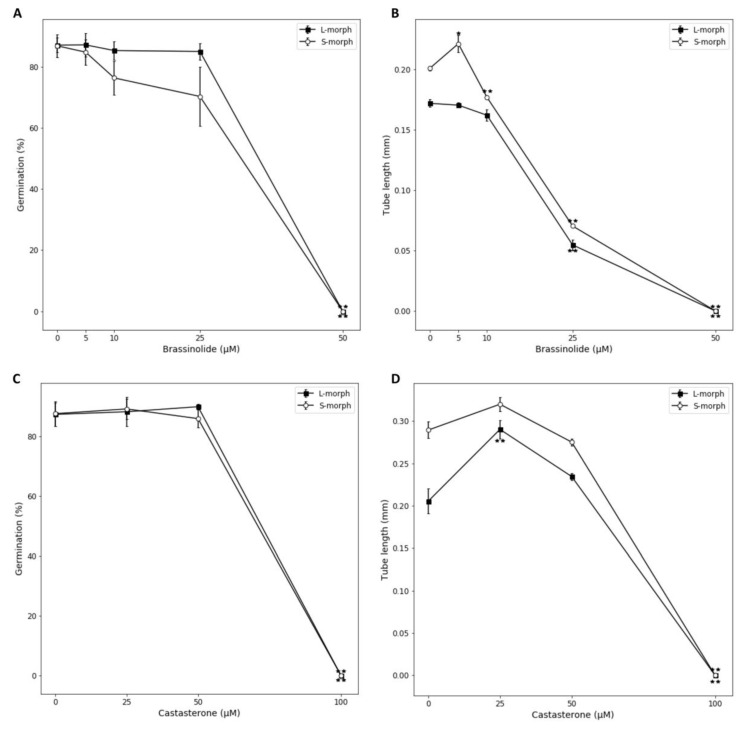
Effects of addition of active BRs on pollen germination and tube growth for S- and L-morph mating types. Effects of addition of brassinolide on pollen germination (**A**) and tube growth (**B**), and addition of castasterone on pollen germination (**C**) and tube growth (**D**). Error bars represent standard error, statistically significant differences between treatments and the control (0 μM) are inidcated: * *p*-values of <0.05 and ** *p*-values of <0.01. calculated using Student’s *t*-test.

In regard to pollen germination, no stimulatory effect was seen on pollen germination on supplementing growth media with BL at any concentration for either morph. A small (but not statistically significant) inhibition of germination was seen for 10 and 25 μM BL treatments of S-, but not L-morph pollen, and both morphs were significantly and dramatically inhibited at 50 μM BL (Figure 5A). This result does not support the hypothesis as the only morph specific inhibition was on S-morph pollen at intermediate BL concentration–counter to the situation where self-incompatibility occurs. For pollen tube growth, BL concentrations above 10 μM were inhibitory for both morphs (Figure 5B). This inhibitory effect of higher concentrations of BRs, on pollen germination and tube growth, has been previously reported for *Arabidopsis* [30]. Intriguingly, however, S-morph pollen showed a small but statistically significant promotion of growth at 5 μM BL, though no inhibitory effect was seen for L-morph pollen at this concentration, and no stimulatory effect on growth was found at any BL concentration for this morph. Representative images of the pollen grown in these treatments are available in Figure S2. Results for castasterone supplementation differed from with those for BL. Though the effects of castasterone on pollen germination were similar, being negligible at low concentrations, but inhibitory at high concentrations in a manner consistent between mating types (Figure 5C), a stimulatory effect on pollen tube growth was observed for both mating types at 25 μM castasterone treatment, but it was only statistically significant for L-morph pollen, above this concentration inhibition of tube growth was again observed in a manner that was consistent between genotypes (Figure 5D).

Though interesting, these results are difficult to interpret. The only clear morph specific effect on pollen was the stimulation of S-morph pollen at 5 μm BL. At this concentration no inhibition of L-morph pollen was observed as was hypothesized if BRs were acting on pollen directly in SI. Further castasterone stimulated growth in both morphs. Self-incompatibility is generally considered to be a result of inhibition of self- rather than promotion of cross-pollen tube growth, but differential inhibition between morphs was not observed at any BR concentration for either BL or castasterone. In *Turnera*, pollen germination occurs in self-pollinations of both morphs, in the S-morph pollen germinates but the tubes generally fail to penetrate through the stigma into the style, in the L-morph the self-incompatible tubes grow a third to half way down the style before ceasing growth [33]. These results suggest that direct stimulation of S-morph pollen by BR could contribute to promoting successful cross-pollination of L-morph pistils, but as the BR concentration at which this might occur (5 μM) has no marked inhibitory effect on self (L-morph) pollen, they do not support BRs acting directly on pollen in SI. Hence, we conclude that SI in *Turnera* is likely mediated by effects downstream of BR regulation of gene expression in the pistils rather than a direct effect on pollen/pollen tubes.

### 2.6. Is the S_H_ Allele in Mhomo-H Present as a Periclinal Chimera?

As noted above, despite bearing mostly homostyle flowers, Mhomo-H periodically, but temporarily, produces flowers with an S-morph phenotype. Phenotypic reversion is not uncommon for mutations associated with transposable elements [34], but there is no obvious mechanism for frequent reversion of point mutations such as that identified in *BAHD* of Mhomo-H (reversion events have continued for more than 20 years since the origin of Mhomo-H). This led us to consider possible mechanisms that did not require physical correction. Close inspection of the sequence chromatograms of all the BAHD sequences at the codon for amino acid 290 revealed that though for Mhomo-S the codon is designated as non-mutant, AAT, a lower secondary peak was present representing a G at position 2 (Figure S3). This suggested the possibility that both wild type and mutant alleles might be present in MhomoS. We hypothesized that the mutation in *TkBAHD* was present as a periclinal chimera, i.e., some cells in the shoot apical meristem possess mutant *TkBAHD*, but others retain the wild type allele. In such a scenario, patterns of cell divisions in the meristem, lateral shoots and/or organs could lead to variable ratios of cells with mutant vs. wild type alleles of *TkBAHD*, potentially leading to the production of mutant long-homostyle or S-morph flowers in the manner observed. It was possible to test this hypothesis as for it to be valid, both mutant and wild type copies of *TkBAHD* need to be present in the original mutant plant (Mhomo-H) as well as plants initiated from it as cuttings (including the revertant Mhomo-S), but sexual progeny from this plant should possess only one of the two alleles as they are derived from a single haploid gamete from the original mutant.

**Figure 6:**
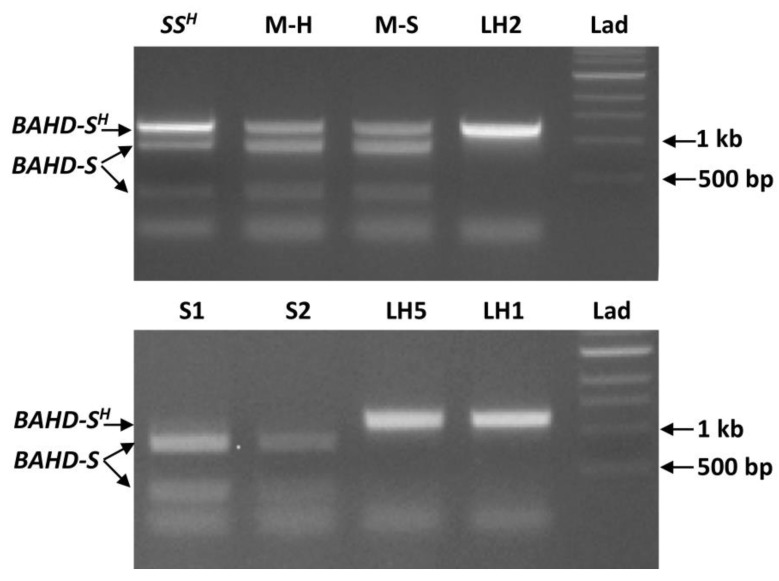
Mfe I Digestion of *TkBAHD* gDNA PCR amplicons. Samples were visualized on an 0.8% agarose gel. Mutant allele (*BAHD-S^H^*) remains a single 1210 bp band, wildtype allele (*BAHD-S*) is cut into 867 bp and 343 bp fragments. DNA samples: upper panel *SS^H^,* control with both wild-type and mutant alleles (MhBRY-9S); M-H, long-homostyle (Mhomo-H); M-S short styled revertent Mhomo-S; LH2, long-homostyle progeny 734H. Lower panel: S-morph progeny, S1 (886S) and S2 (723S) (from the cross of L-morph *T. subulata* x MhBRY-9S) and long homostyle progeny LH2 (734H), and LH1 (859H). LAD: 1 kb ladder.

Fortuitously, the DNA substitution in the mutant form of *TkBAHD* eliminates an Mfe I restriction site, enabling development of a protocol to distinguish between wild type and mutant alleles. Primers (BAHD1F and 1210R, Table S1) were designed to amplify a 1210 bp region of *BAHD* spanning the restriction site, Mfe I digestion of this region of wild type *BAHD* results in two fragments (867 bp and 343 bp), while the mutant form remains undigested as 1210 bp. PCR amplification of *TkBAHD*, followed by digestion with Mfe I and separation of the fragments on agarose gels enabled the allelic content to be determined. A known heterozygous S-morph plant, MhBRY-9S, which possesses both a functional wild type *T. subulata S*-allele and a mutant allele from *T. krapovickasii* (within each nucleus), was included as a control [22]. Both copies of *BAHD* amplified from MhBRY-9S as indicated by bands at 1210 bp (mutant allele), 867 bp and 343 bp (wild type allele) (Figure 6). Similarly, the long-homostyle Mhomo-H mutant and its vegetatively propagated S-morph revertant Mhomo-S also possessed both alleles (Figure 6). In contrast,sexual progeny derived from Mhomo-H (from the cross of *T. subulata* L-morph, D16L x MhBRY-9S see [22]), possessed a single *BAHD* allele, long-homostyle progeny possessing the undigested mutant form of *TkBAHD* as indicated by a single 1210 bp DNA fragment, and S-morph progeny only the wild type allele of *TsBAHD* as indicated by two bands (representatives of 7 progeny tested shown in Figure 6).

The results presented are consistent with mutation of *TkBAHD* having caused the long-homostyle phenotype and support the hypothesis that this mutation arose as a periclinal chimera in Mhomo-H. DNA samples taken from some Mhomo-S floral buds appeared to have a greater percentage of wild type relative to mutant *TkBAHD* than Mhomo-H branches, but the difference was not substantial, possibly because genomic DNA was extracted from whole buds and the location of the cells carrying the mutant allele is critical, that quantitative differences may have been skewed during PCR amplification, or perhaps indicating that a threshold number of cells carrying the wild type allele are necessary for short styled flower morphology.

## 3. Discussion

As the only *S*-locus gene expressed in pistils, *BAHD* has been presumed to control both style length and mating type of the S-morph in *Turnera*. The results presented provide important empirical support for this presumption. Previously we reported that *BAHD* appears to be absent from a long homostyle mutant of *T. scabra* and an X-ray deletion mutant of *T. subulata* [19,22]. We now show it also appears to be absent from the long homostyle species, *T. ulmifolia*. In the sister taxon of *T. ulmifolia* [35], the homostyle species *T. campaniflora, BAHD* was found to be mutated such that the encoded protein is severely truncated. In a spontaneous *T. subulata × T.* *krapovickasii* long homostyle mutant, a single point mutation in a conserved domain of *BAHD* was identified. The original long homostyle mutant plant, Mhomo-H was shown to carry two alleles of BAHD from *T.* *krapovickasii*, one mutated, the other wild-type. Sexual progeny derived from this plant carry a single allele, long homostyles the mutant allele and S-morph progeny the wild type allele. The mutant allele has been used in extensive genetic mapping studies [22,36] and long homostyle progeny are phenotypically stable unlike the original Mhomo-H mutant. These data support the hypothesis that homostyly in Mhomo-H is result of a point mutation in *TkBAHD* and that this mutation occurred in an apical meristem, leading to a chimeric shoot.

Long standing questions are addressed by this study, relating to the mechanism(s) by which BAHD controls style length and physiological mating type. In contrast to the wild type *TkBAHD* allele, the mutant *TkBAHD* allele did not cause dwarfism when expressed ectopically in *Arabidopsis thaliana*, suggesting that the mutant allele has lost much or all of its ability to inactivate BRs and consistent with the conserved GN motif (within which the mutation was identified) being critical to enzyme function [23]. As this mutant allele is associated with long homostyly and self-compatibility, these results suggest that inactivation of BRs by acylation by BAHD pleiotropically mediates both style length and pistil mating type in *Turnera*.

Homomorphic self-incompatibility mechanisms characterized to date employ “lock and key” recognition systems between specific pistil and pollen proteins encoded at *S*-loci, with large numbers of haplotypes that mediate many mating types [37]. It has long been known that distyly is characterized by only two mating types and two haplotypes, but the genetic mechanisms underpinning these mating types have been lacking. As BRs are regulators of cell expansion, inactivation of BRs has a clear potential role in regulating pistil length and indeed the gene controlling pistil length in heterostyly in *Primula* species was recently shown to encode a cytochrome P450, which acts by inactivating BRs [14]. In contrast, how inactivation of BRs in pistil tissues might mediate a self-incompatibility system is less clear. L-morph pistils are predicted to be high in active BR relative to S-morph as a result of the presence of BAHD in S-morph pistils, and our results suggest that the presence of BR in the stigma/style could potentially participate in compatibility of S-morph pollen with L-morph pistils. However, no evidence was found to support the mechanism of self-incompatibility being a direct effect active BRs on pollen germination and tube growth. The alternative way by which BRs might generate a self-incompatibility mechanism is through their effects on transcription. A large number of genes have been reported to be differentially expressed between the pistils of the flower morphs in *Turnera* [31] and the results presented suggest that this differential expression likely underlies self-incompatibility, presumably by changing the physiology of the stigma and stylar transmitting tract. Interestingly this provides support for a long standing hypothesis, that self-incompatibility in heterostyly is more analogous to mechanisms hypothesized for incongruity-interspecies barriers, than homomorphic self-incompatibility systems [38]. The exact nature of the physiological changes that lead to self-incompatibility remain to be investigated, but the fact that *CYP734A50*, which controls pistil characters in heterostylous *Primula* species also encodes a BR inactivating enzyme [14] suggests that these *S*-genes may be co-opting a general pathway present in many plant species. An analogous situation has been reported for the *S*-genes of *Papaver*, which can be used to recapitulate the *Papaver* homomorphic self-incompatibility in *Arabidopsis* by plugging into the components of an endogenous pathway [39].

Though this is, as far as we are aware, the first report documenting a periclinal meristem mutation leading to homostyly, it is not inherently surprising that meristems can be chimeric for mutant and wild type alleles and as a result produce either wild type or homostyle flowers depending on their allelic composition. Being able to catch and document this process occurring in a woody perennial that can be maintained by cuttings was, however, immensely fortuitous. The genus *Turnera* possesses a considerable number of long homostyle species, given that mutation of *BAHD* pleiotropically changes both morphology and physiology to create a self-compatible long homostyle, the path by which Mhomo-H arose is likely to be common to many of those species.

There are potentially broader phylogenetic implications for this research to the evolution of heterostyly. BR homeostasis is tightly regulated in plants and a considerable number of genes and molecular mechanisms that inactivate BRs have been identified in *Arabidopsis* [40]. Heterostyly has evolved independently many times, given the number of different genes that can inactivate BRs and that any one has the potential to have a pleiotropic effect on pistil mating type morphology and physiology, we speculate that recruitment of these genes to *S*-loci to control pistil characters by BR inactivation has the potential to be of broad phylogenetic significance to the evolution of heterostyly across the flowering plants.

## 4. Materials and Methods

### 4.1. Plant Material

Plants were grown in greenhouse conditions at York and Washington State Universities. The long-homostyle mutant, Mhomo-H has been previously described [22]. This plant arose as a sport-shoot on an otherwise short styled *T. krapovickasii* × *T. subulata* diploid hybrid plant and has been maintained by vegetative propagation. Progeny carrying the mutant *S_H_* allele were previously generated for recombination mapping of the *S*-locus [22]. Briefly, Mhomo-H was crossed to an S-morph *T. subulata*, and an S-morph progeny plant, MhBRY-9S, was obtained that was heterozygous at the S-locus, *SS_H_*, with the *S*-allele derived from *T. subulata*, and the mutant *S_H_*-allele from *T. krapovickasii*. MhBRY-9S was then crossed to an L-morph plant of *T. subulata* yielding numerous (>2000) progeny segregating for approximately equal frequencies of homostyle and S-morph progeny [22]. Progeny from this cross were used for various analyses, where the homostyle progeny (233H, 275H, 734H, 859H, 867H) are genotypically *S_H_s*, carrying the mutant *S_H_*-allele and flanking DNA of *T. krapovickasii* (and the recessive *s*-allele of *T. subulata*) while the S-morph progeny (723S and 886S) are genotypically *Ss*, both alleles (and flanking DNA) having been derived from *T. subulata*. We also used a homostyle plant that was homozygous, *S_H_S_H_*_,_ previously generated by selfing homostyle progeny plant 233H and screening progeny for *T. krapovickasii* associated genetic markers flanking the *S*-locus as well as the absence of markers associated with the recessive *s*-haplotype of *T. subulata* [41].

### 4.2. Genomic PCR

A rapid small-scale Mini-CTAB DNA extraction protocol, outlined by Labonne et al. [36] and modified from Doyle and Doyle [42], was used to isolate genomic DNA. An intermediate-sized (~5 mm long) flower bud was ground in 100 µL CTAB isolation buffer [2% *w*/*v* CTAB (Sigma-Aldrich, St. Louis, MO, USA), 1.4 M NaCl, 0.2% *v*/*v* 2-mercaptoethanol, 20 mM EDTA,100 mM Tris-HCl (pH 8.0)] on ice. An additional 400 µL of CTAB isolation buffer was added to each sample before incubating at 60 °C for 30 min with occasional mixing. In total, 500 μL of chloroform/isoamyl alcohol (24:1) was added to each sample, which were mixed and then centrifuged for 5 min at 15,000× *g*. After transferring the supernatant to new microcentrifuge tubes, nucleic acids were precipitated by adding 400 μL cold isopropanol and centrifuging for 5 min at 15,000× *g.* The supernatant was removed and the pellets washed with 500 µL wash buffer (76% Ethanol, 10 mM ammonium acetate). The wash buffer was removed, and the pellet air-dried before resuspending the DNA in 200 μL of TE (10 mM Tris-HCl pH 8, 1 mM EDTA). RNA was removed by incubation at 37 °C in the presence of RNase A (10 μg/mL). PCR reactions were performed using ~100 ng of genomic DNA, 2 μL of 10 pmol/μL of each primer, 4 μL ddH_2_O, and 10 μL Quick-Load**^®^** Taq 2X Master Mix (New England BioLabs, Ipswich, MA, USA). Cycling parameters: 5 min denaturation (94 °C) followed by 35 cycles of 30 s at 94 °C, 30 s at 58 °C, 30 s at 68 °C, followed by 5 min at 68 °C. For amplicons greater than 1 kb, annealing temperature and extension times were increased as appropriate (60 s for >1 kb). After completion, samples were held at 4 °C, run on agarose gels (see below) or frozen at −20 °C for later use. DNA sequencing was performed at the Génome Québec Innovation Centre using an Applied Biosystems 3730xl DNA Analyzer (Foster City, CA, USA). Sequences were assembled using Sequencher version 5.4.6 DNA sequence analysis software (Gene Codes Corporation, Ann Arbor, MI, USA).

### 4.3. RT-PCR

Plant tissue (up to 100 mg styles where available) was ground in liquid nitrogen. Total RNA was isolated using the Rneasy Plant Mini Kit (Qiagen, Germantown, MD, USA) following the manufacturer’s protocol coupled with “on column” DNA digestion using Rnase free Dnase (Qiagen, Germantown, MD, USA). RNA was quantified using a nanodrop 2000 spectrophotometer (Thermo Scientific, Waltham, MA, USA) and cDNA synthesized using 300–1000 ng total RNA with oligo-dT priming and a ProtoScript II First Strand cDNA Synthesis Kit (New England Biolabs, Ipswitch, MA, USA), following the manufacturer’s protocol. cDNA (1 µL) was used in RT-PCR (following PCR protocol below) to detect expression of genes of interest using gene-specific primers (Table S1). PCR products were visualized on 0.8–1.5% agarose gels.

### 4.4. Purification and Mfe I Digestion of BAHD Amplicons

RT-PCR was performed on style cDNAs using the primers BAHD1F and BAHD1210R (see Table S1) to generate a 1210 bp amplicon. PCR products were run on 1.0% agarose gels, and following visualization the amplicons excised and purified using the EZ-10 Spin Column PCR Products Purification Kit according to the manufacturer’s instructions (BioBasic, Markham, ON, Canada). In total, 6 μL (~600 ng of DNA) of gel-purified amplicon was digested with Mfe1-HF (New England Bio Labs, Ipswitch, MA, USA) in a total volume of 25 μL for ~3 h at 37 °C. An additional 1 μL (20 U) of restriction enzyme was added after 2 hrs to promote complete digestion. Digests were visualized on 1% agarose gels.

### 4.5. Plasmid Construction and Generation and Analysis of Transgenic Lines

The polymerase chain reaction (PCR) was used to amplify the *TkBAHD* coding sequence from genomic DNA using Accuzyme™ proofreading Taq DNA polymerase (Bioline, Memphis, TN, USA). The gene-specific primers used were BAHD-kpnF and BAHD-bhR (Table S1) at 58 °C annealing temperature. PCR products were purified using a Zymoclean™ Gel DNA Recovery Kit according to the manufacturers’ instructions (ZymoResearch, Irvine, CA, USA), ligated into pGEM-T^®^ Easy vector (Promega, Madison, WI, USA) and transformed into *E. coli*. Plasmid DNA was purified using the ZR Plasmid Miniprep-Classic (ZymoResearch, Irvine, CA, USA), ligated into pGEM-T^®^ Easy vector (Promega, Madison, WI, USA), and sequenced. *Bam*H1 and *Kpn*1 restriction sites were added using primers BAHD_kpn1F and BAHD_bh1R (Table S1) to facilitate cloning behind the CaMV-35S promoter in the binary vector pCHF3) [29]. Plasmid constructs were transformed into *Agrobacterium* strain GV3101 and transformed into *A. thaliana* ecotype Col-0 using the floral dip method [43]. Screening of transgenic seedlings and RT-qPCR analyses were performed as described in [20]. Sequences for the gene specific primers used for BAHD (BAHDqF and BAHDqR) and the actin control (ActinqF and ActinqR are listed in Table S1).

### 4.6. Pollen Germination and Growth Assays

Pollen from flowers of greenhouse grown *Turnera joelii* was added directly to 500 μL of pollen germination media (PGM; 0.01% H_3_BO_3_, 0.02% MgSO_4_, 0.07% CaCl_2_, 20% PEG4000, 12% sucrose, 10 mM MES-KOH, pH 6.0) in a 15 mL tube which was placed horizontally on a tilting platform and cultured at room temperature (25–30 °C) for 2.5 h. For brassinolide treatments, brassinolide was added directly to the PGM to final concentrations of 0 μM, 5 μM, 10 μM, 25 μM or 50 μM. For castasterone treatments, castasterone (0 μM, 25 μM, 50 μM, 100 μM, or 250 μM final concentrations) was added directly to the PGM prior to the addition of pollen. In total, 200 pollen tubes were measured from each replicate for each treatment. Tube length was measured using ImageJ2 [44]. Germination was scored for all pollen grains present for each image for each replicate.

## Data Availability

Gene sequences described in this work can be accessed in GenBank.

## Supplementary Materials

The following are available online at https://www.mdpi.com/article/10.3390/ijms221910603/s1.
