# Supplementary material for: Pistil Mating Type and Morphology Are Mediated by the Brassinosteroid Inactivating Activity of the S-Locus Gene BAHD in Heterostylous Turnera Species
Source: Int J Mol Sci. 2021 Sep 30;22(19):10603. doi: 10.3390/ijms221910603 (PMC8509066; doi:10.3390/ijms221910603)
Supplement: Supplementary file 1 [file ijms-22-10603-s001.zip › Figure S1.pdf]

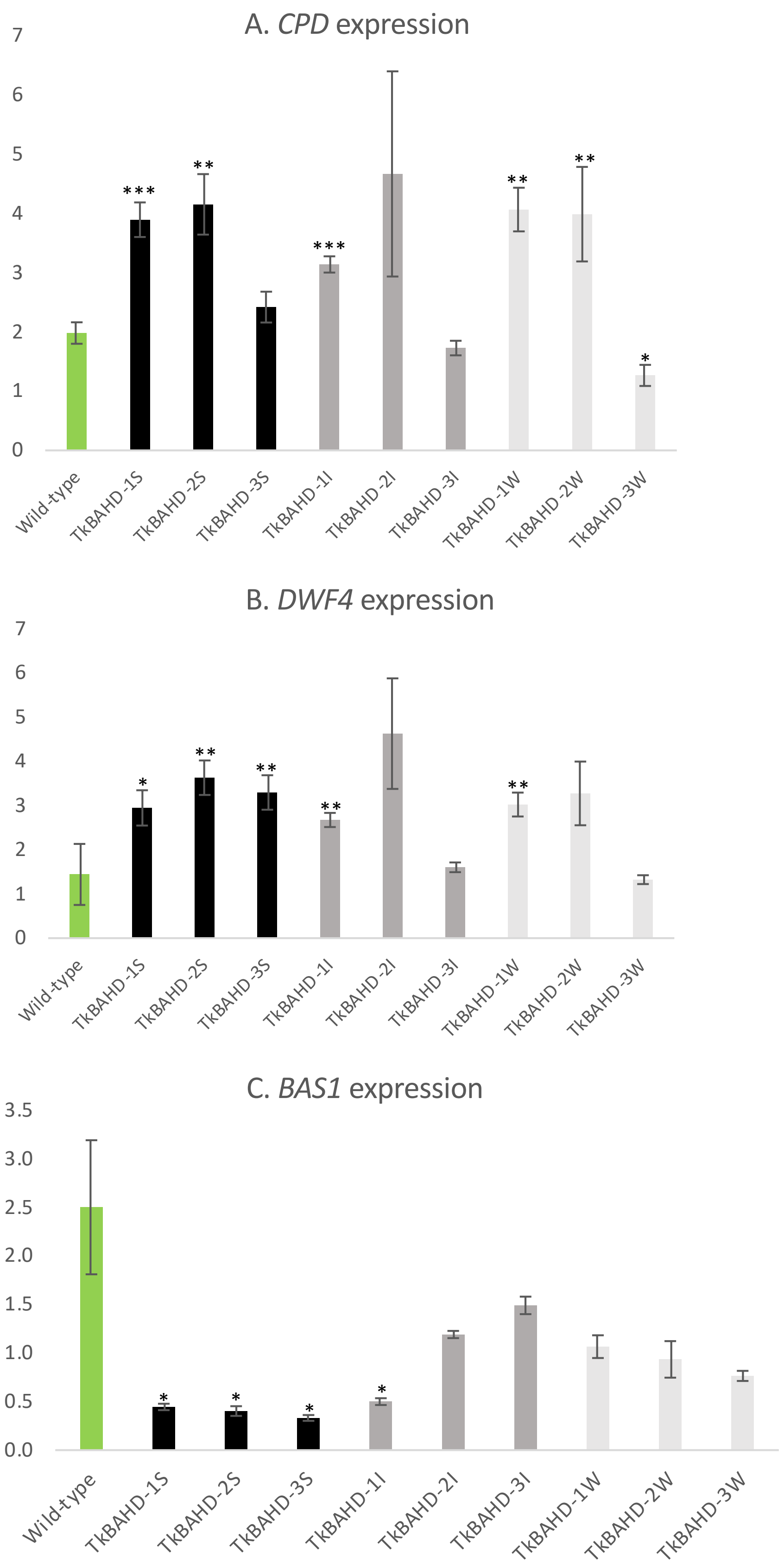

Figure S1. RT-qPCR of CPD, DWF4 and BAS1 in transgenic Arabidopsis lines expressing *TkBAHD.* Expression levels (relative to actin) of *endogenous CPD, DWF and BAS1* in three *35S::*TkBAHD lines (each divided into S, I, and W dwarf categories) and wild-type samples. Expression was normalized to the actin 8 (At1g4920) housekeeping gene. Error bars represent standard error. *p*-values comparing *35S::TsBAHD* lines to the wild type were calculated using Student's t-test comparing each line to the wild-type expression * $p < 0.05$, ** $p < 0.01$, *** $p < 0.001$. (Details of the method and primers used were as previously described [20]).
