# Supplementary material for: Pistil Mating Type and Morphology Are Mediated by the Brassinosteroid Inactivating Activity of the S-Locus Gene BAHD in Heterostylous Turnera Species
Source: Int J Mol Sci. 2021 Sep 30;22(19):10603. doi: 10.3390/ijms221910603 (PMC8509066; doi:10.3390/ijms221910603)

Images pollen tubes for BL treatments

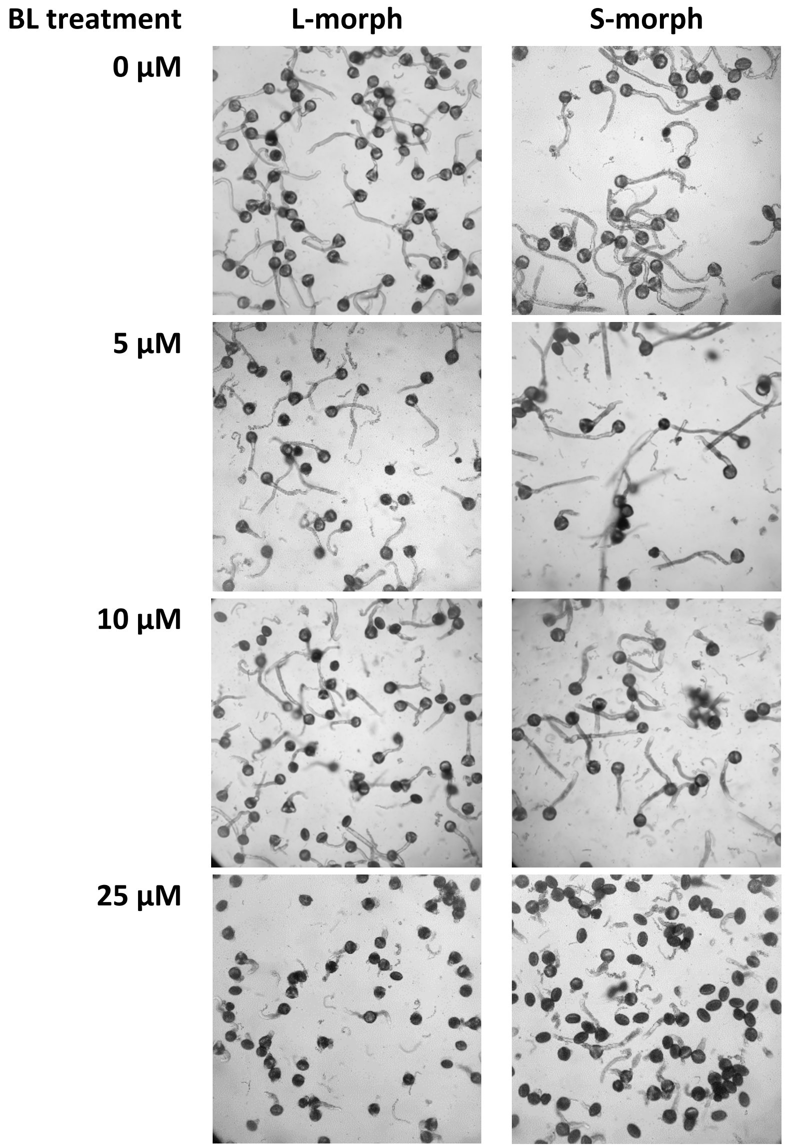

Violin plot of pollen tube growth data for BL treatments

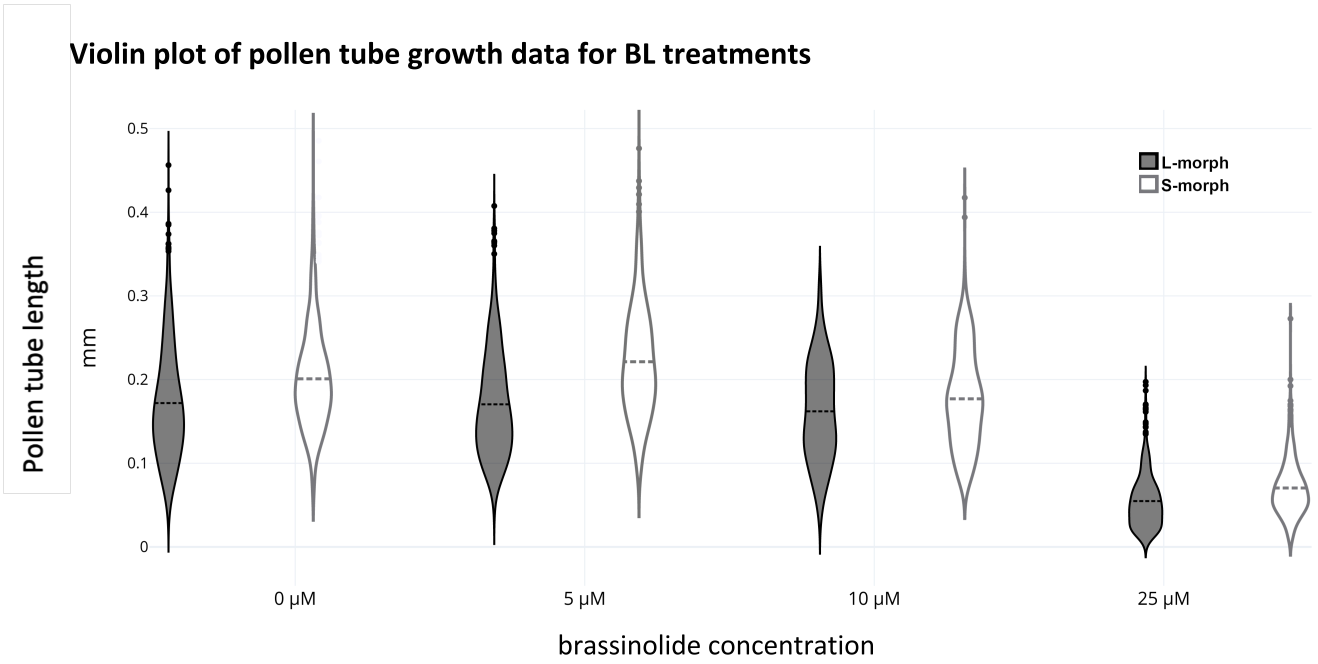

Images pollen tubes for BL treatments

CASTASTERONE TREATMENT | **L-morph** | **S-morph**

0 µM

25 µM

50 µM

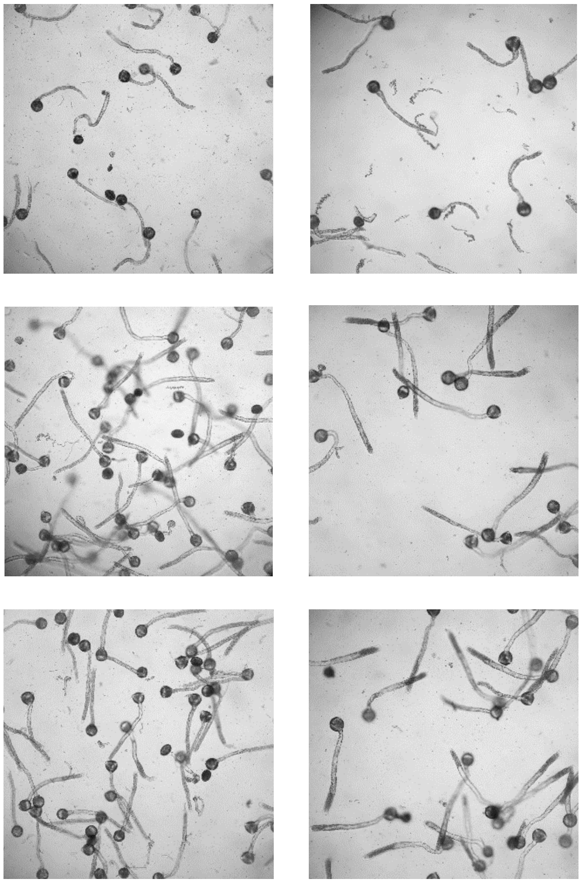

Violin plot of pollen tube growth data fro BL treatments

Violin plot of pollen tube growth data for Castasterone treatments

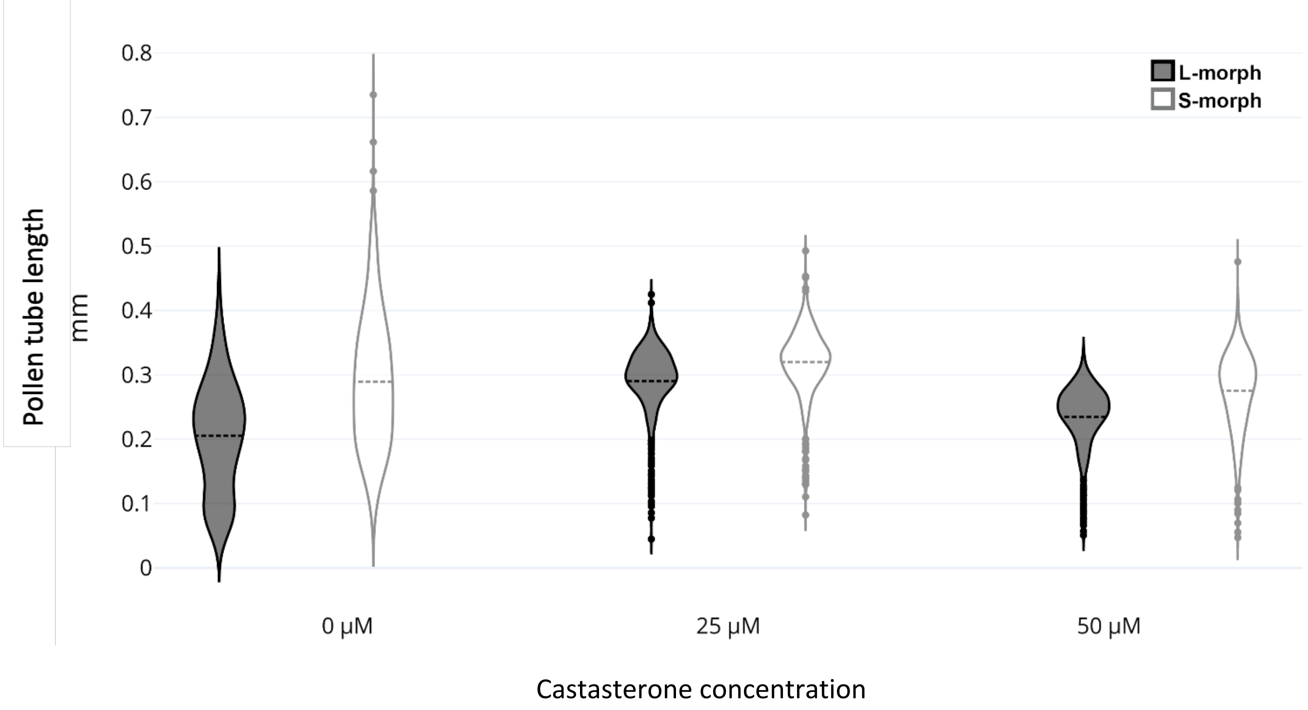

Supplement: Supplementary file 1 [file ijms-22-10603-s001.zip › Figure S2 .pdf]
