# Supplementary material for: Pistil Mating Type and Morphology Are Mediated by the Brassinosteroid Inactivating Activity of the S-Locus Gene BAHD in Heterostylous Turnera Species
Source: Int J Mol Sci. 2021 Sep 30;22(19):10603. doi: 10.3390/ijms221910603 (PMC8509066; doi:10.3390/ijms221910603)
Supplement: Supplementary file 1 [file ijms-22-10603-s001.zip › Table S1 Primers.pdf]

Primer	Sequence 5’-3’
BAHD-36F	AGCCTCCAGCAACAAAGTAA
BAHD1F	ATGGAAGTTGAGATCACGCTG
BAHD664F	GCAAAGATACGCATGCTCAA
BAHD1179F	AGATACAAGGGATGCTGCCG
BAHD522R	CGATCCGCTCCAGCATTTGA
BAHD1003R	GCAAACCCACAAATTGCTTT
BAHD1022R	GCAAACCCACAAATTGCTTT
BAHD1210R	ATGCTTCTATTCCGGCAGCA
BAHD1513R	ACGGGGCTATCGGAGAAAGT
TUB1F	CAGCTGGAAAGGATCAATGTTTA
TUB1R	GTTCTTGGCATCCCACATTT
BAHD-kpnF	GGTACCTTAAGATATGGAAGTTGAGAT
BAHD-bhR	GGATCCCAAAAGCATGATTCTG
BAHD-qF	ACAGCTCCTGCCTGCA
BAHD-qR	CAACTCAACTGAGGAGGGTCT
Actin-qF	GGTCGTACAACCGGTATTGT
Actin-qR	GATAGCATGTGGAAGTGAGAA

- Primer names indicated in red were used for internal sequencing of BAHD genes
