# Supplementary figures and images for: Pistil Mating Type and Morphology Are Mediated by the Brassinosteroid Inactivating Activity of the S-Locus Gene BAHD in Heterostylous Turnera Species
Source: Int J Mol Sci. 2021 Sep 30;22(19):10603. doi: 10.3390/ijms221910603 (PMC8509066; doi:10.3390/ijms221910603)

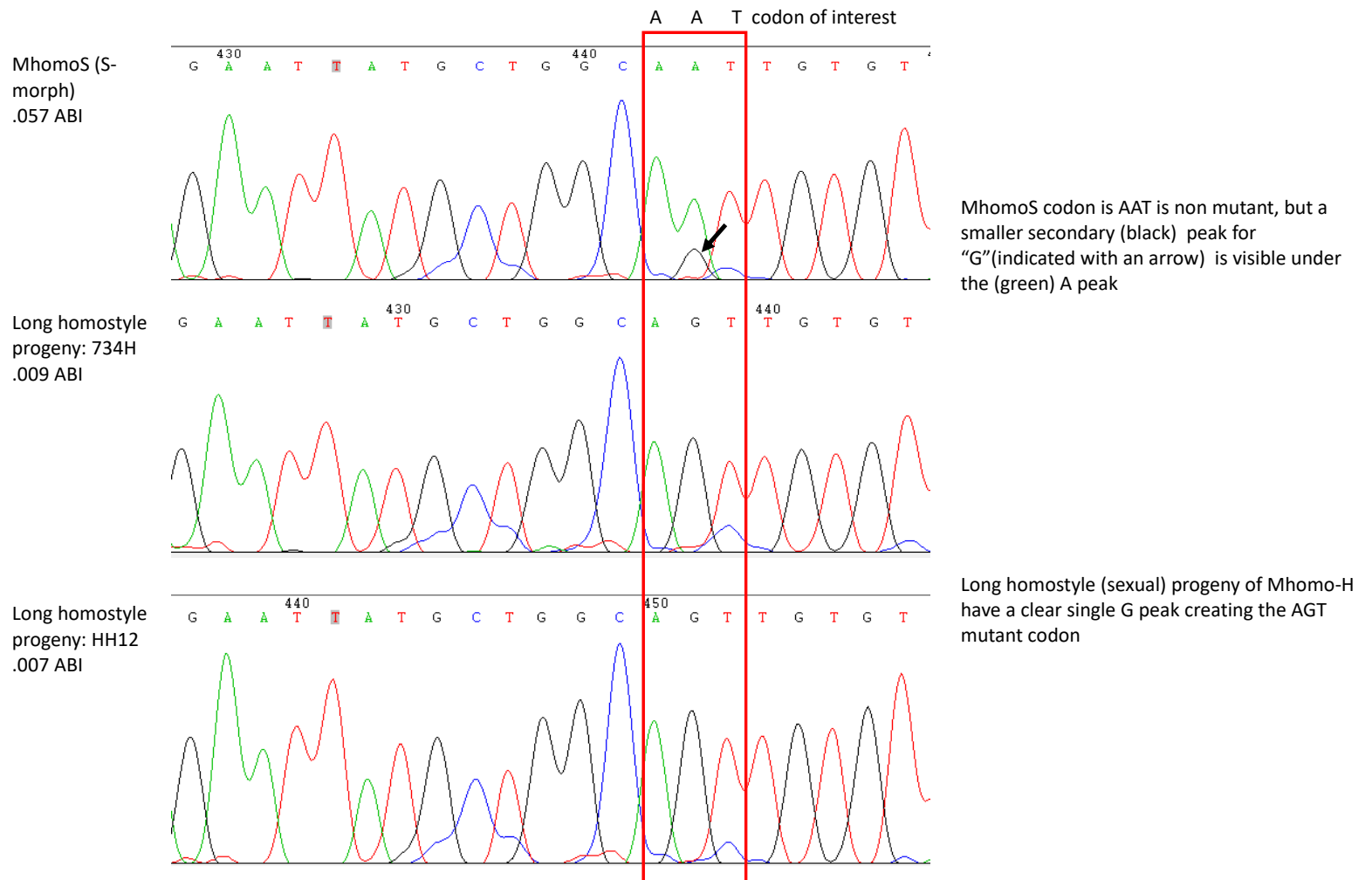

Supplement: Supplementary file 1 [file ijms-22-10603-s001.zip › Figure S3.pdf]
